# A Novel Murine Chronic Obstructive Pulmonary Disease Model and the Pathogenic Role of MicroRNA-21

**DOI:** 10.3389/fphys.2018.00503

**Published:** 2018-05-04

**Authors:** Shengyang He, Liqiu Li, Shenghua Sun, Zhengpeng Zeng, Junjuan Lu, Lihua Xie

**Affiliations:** Department of Respiratory Medicine, The Third Xiangya Hospital of Central South University, Changsha, China

**Keywords:** COPD, animal model, microRNA-21, inflammation, biomarker

## Abstract

Chronic obstructive pulmonary disease (COPD) is a multi-pathogenesis chronic lung disease. The mechanisms underlying COPD have not been adequately illustrated. Many reseachers argue that microRNAs (miRs) could play a crucial role in COPD. The classic animal model of COPD is both time consuming and costly. This study proposes a novel mice COPD model and explores the role of miR-21 in COPD. A total of 50 wide-type (WT) C57BL/6 mice were separated into five euqlly-sized groups—(1) control group (CG), (2) the novel combined method group (NCM, cigarette smoke (CS) exposure for 28 days combined with cigarette smoke extract (CSE) intraperitoneal injection), (3) the short-term CS exposure group (SCSE, CS exposure for 28 days), (4) the CSE intraperitoneal injection group (CSEII, 28 days CSE intraperitoneal injection), and (5) the long-term CS exposure group (LCSE, CS exposure).The body weight gain of mice were recorded and lung function tested once the modeling was done. The pathological changes and the inflammation level by hematoxylin eosin (H&E) staining and immunohistochemical staining (IHS) on the lung tissue sections were also evaluated. The level of miR-21 in the mice lungs of the mice across all groups was detected by RT-qPCR and the effects of miR-21 knock-down in modeled mice were observed. The mice in LCSE and NCM exhibited the most severe inflammation levels and pathological and pathophysiological changes; while the changes for the mice in SCSE and CSEII were less, they remained more severe than the mice in the CG. The level of miR-21 was found to be negatively correlated with lung functions. Moreover, knocking miR-21 down from the modeled mice, ameliorated all those tested COPD-related changes. Our novel modeling method detected virtually the same changes as those detected in the classic method in WT mice, but in less time and cost. Further, it was determined that the level of miR-21 in the lungs could be an indicator of COPD severity and blocking functions of miR-21 could be a potential treatment for early stage COPD.

## Background

Continued development and industrialization has given rise to the expansion of various chronic diseases which, in turn, have significantly impacted families across the globe. Chronic obstructive pulmonary disease (COPD) is now considered to be the fourth leading cause of morbidity and mortality worldwide, with epidemiologists ranking it third, behind heart disease and stroke, as being a contributor to human death globally (Gautam and O'Toole, 2016).

COPD, which features irreversible and limitations on persistent airflow, is generally considered to be a preventable and treatable disease. For COPD patients who are in a stable phase, noxious particles, and gases could induce acute exacerbation of the disease and result in it becoming irreversible (Brusselle and Bracke, 2015). Smoking is generally accepted as the most important, independent risk factor for COPD; the specific pathogenesis and mechanism are, however, inadequately illustrated (Vogelmeier et al., 2017). Building an animal model is believed to be one of the more effective and efficient ways to further study this disease. Many different animals have been utilized in making COPD models, including rodents, canines, goats, and monkeys (Wright and Churg, 2010). Mice are generally considered to be the best choice, given that they share a similar genetic background to that of humans, they're easy to breed, and reproduce quickly. The most recognized method adopted by researchers in studying COPD is the long-term cigarette smoke (CS) exposure method (Wright et al., 2008; Givi et al., 2013). However, this method costs a lot to implement and takes a long time which, in turn, slows down the whole process of research. Through the efforts of many researchers more COPD modeling methods have been suggested and demonstrated. These alternate methods include: intranasal instillation of both elastase and LPS (Pera et al., 2014), only sulfur dioxide exposure (Wagner et al., 2006), inhalation of ovalbumin dry powder (Misaka et al., 2009), intravenous injection of hyaluronidase (Tazaki et al., 2006), genetic manipulation (Baron et al., 2012), intraperitoneal injection with xenogeneic endothelial cells (Taraseviciene-Stewart et al., 2005), and intraperitoneal injection with CSE (He et al., 2013). All of these alternate methods are either too complicated or require too much time. More importantly, the pathogeneses of these alternate methods may not be clinically relevant and potentially lead to bias conclusions.

MicroRNAs (miRs) are expressed in an endogenous way by cells, functioning as a non-coding role but regulating gene expression at the post-transcriptional level. Briefly, miRs, by binding to the 3′ untranslated regions of the target mRNAs' sequence, cause inhibition or degradation and thus eventually interfere in the expression process (Bartel, 2004; Di Leva and Croce, 2013). MiR-21, being one of the most multi-functional miRs, is usually investigated in tumor related diseases (Selcuklu et al., 2009). However, increasingly intensive studies involving miR-21 have led researchers to conclude that miR-21 also plays an indispensable role in inflammation (Sheedy, 2015; Li et al., 2017; Venugopal et al., 2017).

Our immune system, comprising numerous and different types of immune cells, is the main source of inflammation. MiR-21 was found in the progenitors of immune cells and its expression level was enhanced during the mature and activation periods of such immune cells. Regardless of whether they were mast-cells, neutrophils, or activated T-cells of various lineages, high levels of miR-21 were observed in all of them (Lu et al., 2005; Monticelli et al., 2005; Cobb et al., 2006; Wu et al., 2007). Such observations suggest miR-21 might be related to the activation and severity of inflammation response.

For some inflammatory diseases, particularly allergic airway inflammation (Moschos et al., 2007; Lu et al., 2009), studies have noted increased levels of miR-21 in tissue compared with control group levels. In other diseases, like osteoarthritis and atherosclerosis, enhanced levels of miR-21 have been observed (Raitoharju et al., 2011; Zhang et al., 2014). These other diseases share one point in common—a massive infiltration of immunocytes in the inflammation tissue. In the same manner, our group previously found that macrophages, mainly the M2 phenotype, tend to deposit in the alveoli of COPD mice, which could be related to TGF-β/Smad pathway (He et al., 2017); this pathway was concerned to be a target of miR-21 (Davis et al., 2008). To confirm that the rise of miR-21 is not only local but also circulatory, our group analyzed the miRs expression profile in COPD by microarray and confirmed by RT-qPCR that miR-21 is ascendant in the serum of COPD patients and asymptomatic heavy smokers (Xie et al., 2014). This finding is in line with the concept that “COPD is a systematic inflammatory syndrome” as presented by Fabbri (Fabbri and Rabe, 2007), indicating that miR-21 might be valuable for evaluating the development of COPD in heavy smokers.

Certainly, proposing a cost effective and time efficient method with remarkable effects might be of great significance; the practicability of miR-21 working as an indicator of COPD severity needs further confirmation. In this study, we proposed a novel COPD modeling method and compared it with other reported methods to evaluate its effects. We introduced miR-21 as an indicator of COPD severity and confirmed the pathogenic role of miR-21 in COPD.

## Materials and methods

### Animals and modeling methods

A total of 50 6-week-old wide-type (WT) C57BL/6 mice were bought from the Vital River Laboratory Animal Technology Co., Ltd (Beijing, China). They were divided into five groups of 10 mice each and subjected to five different modeling methods, as follows: (1) control group (CG), (2) novel combined method group (NCM, cigarette smoke (CS) exposure for 28 days combined with cigarette smoke extract (CSE) intraperitoneal injection), (3) short-term CS exposure group (SCSE, CS exposure for 28 days), (4) CSE intraperitoneal injection group (CSEII, 28 days CSE intraperitoneal injection), and (5) long-term CS exposure group (LCSE, CS exposure). Briefly, the CG mice were maintained in fresh air with no special treatment and in days 1, 12, 23 they were given an intraperitoneal injection of phosphate-buffered saline (PBS; 0.3 ml/20 g). The NCM mice were given CS exposure in a sealed box with a ventilation hole for two cycles per day and 28 days in total, except for days 1, 12, 23 and over these 3 days the mice were given an intraperitoneal injection of 100% CSE solution (0.3 ml/20 g; preparation method is described in the following part). The SCSE mice were given CS exposure in a sealed box with a ventilation hole for two cycles per day and 28 days in total. The CSEII mice were maintained in fresh air and given an intraperitoneal injection of 100% CSE solution on days 1, 12, 23. The LCSE mice were given CS exposure in a sealed box with a ventilation hole for two cycles per day and 6 months (180 days) in total.

The miR-21 knock-out (miR-21^−/−^) mice were purchased from the University of Texas, Southwestern Medical Center. Modeling method is in accordance with the NCM. This study was approved by the Institutional Review Board of Central South University following the guiding principles for research involving animals and humans (World Medical and American World Medical Association American Physiological Society., 2002).

The LCSE and CG mice were sacrificed after undergoing lung function tests at day 181 of modeling; mice in the other groups, and miR-21^−/−^ mice were sacrificed at day 29 of modeling, after undergoing lung function tests.

### Preparation of CSE

In short, each non-filter Furong cigarette (tar: 13 mg, nicotine: 1.0 mg, carbon monoxide: 14 mg/ cigarette; China Tobacco Hunan Industrial Co. Ltd., Changsha, China) was burned, and all the smoke was dissolved in 10 ml PBS as the 100% CSE. Before injection, a 0.22-μm pore filter (Thermo Fisher Scientific, Waltham, MA, USA) were utilized to filter particles and bacteria away from CSE. The solution was utilized in 30 min after preparation.

### Lung function tests

A small animal lung function instrument (PLY3211; Buxco Research Systems, Wilmington, NC, USA) provided by the School of Basic Medical Science of Central South University (Changsha, China), was applied for lung function measurement of all mice enrolled in the present study before sacrifice. Data of mice who underwent a complete lung function test were collected for further analyses.

### Hematoxylin and eosin (H&E) staining of lung tissue

Every lung tissue section was from the right middle lobe of each mouse and was inflated by 4% paraformaldehyde at a constant pressure of 25 cmH2O and then fixed with 4% paraformaldehyde for 24 h. The fixed lung tissue was embedded by paraffin (Sigma-Aldrich Co., St Louis, MO, USA) and sectioned into 4-μm sections for further staining with H&E (Sigma-Aldrich Co.). The mean linear intercept (MLI), mean alveolar septal thickness (MAST) and destructive index (DI) were collected from the H&E staining sections to evaluate the emphysematous changes of the mice as previously described in another study (Lu J. et al., 2017). We enrolled five lung tissue sections from each mouse who underwent a complete lung function test and randomly chose five 100X fields from each section. MLI is a measurement of mean interalveolar septal wall distance, which is widely used to examine alveolar space size. As previously described (Choe et al., 2003), the MLI was measured by dividing the length of a line drawn across the lung section by a total number of intercepts counted within this line at 100X magnification (the bronchial regions were not included). The DI was calculated by dividing the defined destructive alveoli by the total number of alveoli (Destructive alveolus was defined if at least one of the following alveoli was observed: alveolar wall defects, intraluminal parenchymal rags in alveolar ducts, obviously abnormal morphology, and typically emphysematous changes). The MAST was the thickness of the alveolar septum, including alveolar epithelial tissue and alveolar interval groups.

### Immunohistochemical staining (IHS)

We conducted the IHS to detect the expression levels of CD3, CD68, and MPO (representing lymphocytes, macrophages and neutrophils respectively) in lung tissue by utilizing the specific antibodies of CD3 (diluted 1:100; Proteintech, Chicago, IL, USA), CD68 (diluted 1:100; Proteintech, Chicago, IL, USA) and MPO (diluted 1:100, R&D Systems, Inc., Minneapolis, MN, USA). The 4 μm paraffin-embedded lung tissue sections as described above were utilized for IHS. To block endogenous peroxidase activity, these sections were incubated with 3% H_2_O_2_ for 30 min at room temperature. Then, the lung tissues were incubated overnight at 4°C with all the first antibodies mentioned above. The sections were then washed carefully by PBS and incubated with the appropriate horseradish peroxidase-conjugated secondary antibodies for 15 min at 37°C. Then, the targets were visualized by diaminobenzidine (DAB, Sigma-Aldrich Co.) and counterstained with hematoxylin. We enrolled five lung tissue sections from each mouse who underwent a complete lung function test and randomly chose five 400X fields from each section to do the further analyses. The number counting process of positive cells in each 400X field was handled by a professional pathologist in a double-blind way.

### Real-time reverse transcription polymerase chain reaction (RT-PCR)

The total RNA were isolated by TRIzol reagent(Thermo Fisher Scientific, Waltham, MA, USA) following the manufacturer's protocol. Then, the reverse transcription process is carried out by PrimeScript™ RT Reagent Kit (TaKaRa, Dalian, Liaoning, China). Further, the relative expression level of mRNA was assessed by SYBR Green PCR Master Mix (Bio-Rad, Laboratories Inc., Hercules, CA, USA) and quantified by two-step quantitative real-time polymerase chain reaction (PCR; Applied Biosystems, Carlsbad, CA, USA). U6 was used as an internal control, and 2^−ΔΔCT^ method was employed to calculate the expression of miR-21. The primer of miR-21 is Bulge-Loop™ (RiboBio, Guangzhou, Guangdong, China).

### Statistical analysis

All the data were analyzed by SPSS 22.0 statistical software (IBM Corporation, Armonk, NY, USA) and are presented as mean ± standard error of the mean (SEM). The statistical difference between groups were examined using Student's unpaired *t*-test and a one-way ANOVA test. And we employed the Pearson's correlation coefficient between groups to analyze the correlation. *p* < 0.05 was considered as statistically significant.

## Results

### Models evaluation

**Figure 1:**
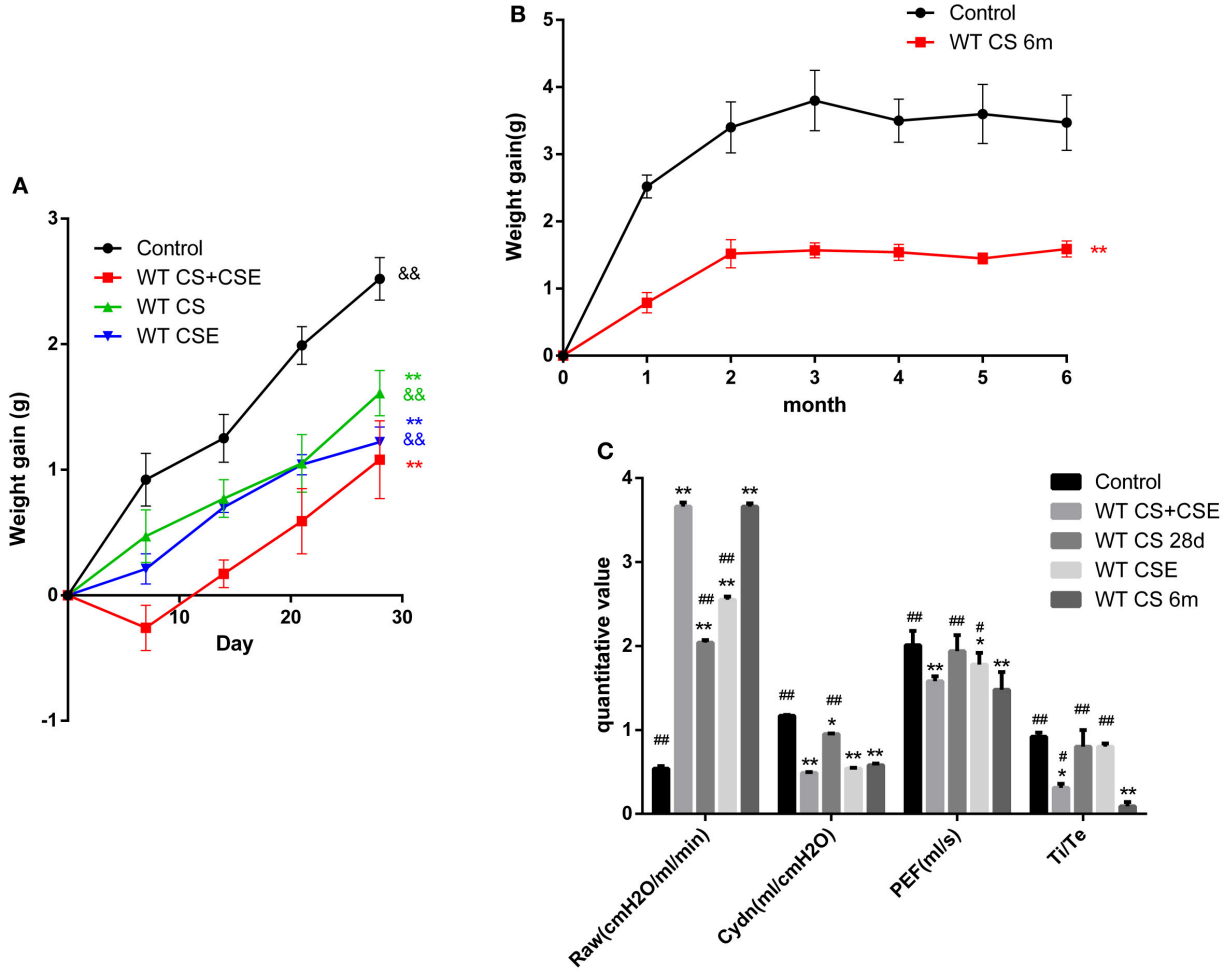
Changes of body weight and lung functions of WT mice models by different methods. **(A,B)**, trends of mice body weight in modeling days of different group. **(C)** lung functions of mice in different group. Raw, Airway resistance; Cdyn, lung dynamic compliance; PEF, peak expiratory flow; Ti /Te Inspiratory time/expiratory time. ^*^Compared with control group, *p* < 0.05; ^**^compared with control group, *p* < 0.001; ^#^compared with WT CS 6m group, *p* < 0.05; ^##^compared with WT CS 6m group, *p* < 0.001. ^&^compared with WT CS+CSE group, *p* < 0.05; ^&&^compared with WT CS+CSE group, *p* < 0.001 Error bars: mean ± SEM. *n* = 5.

**Figure 2:**
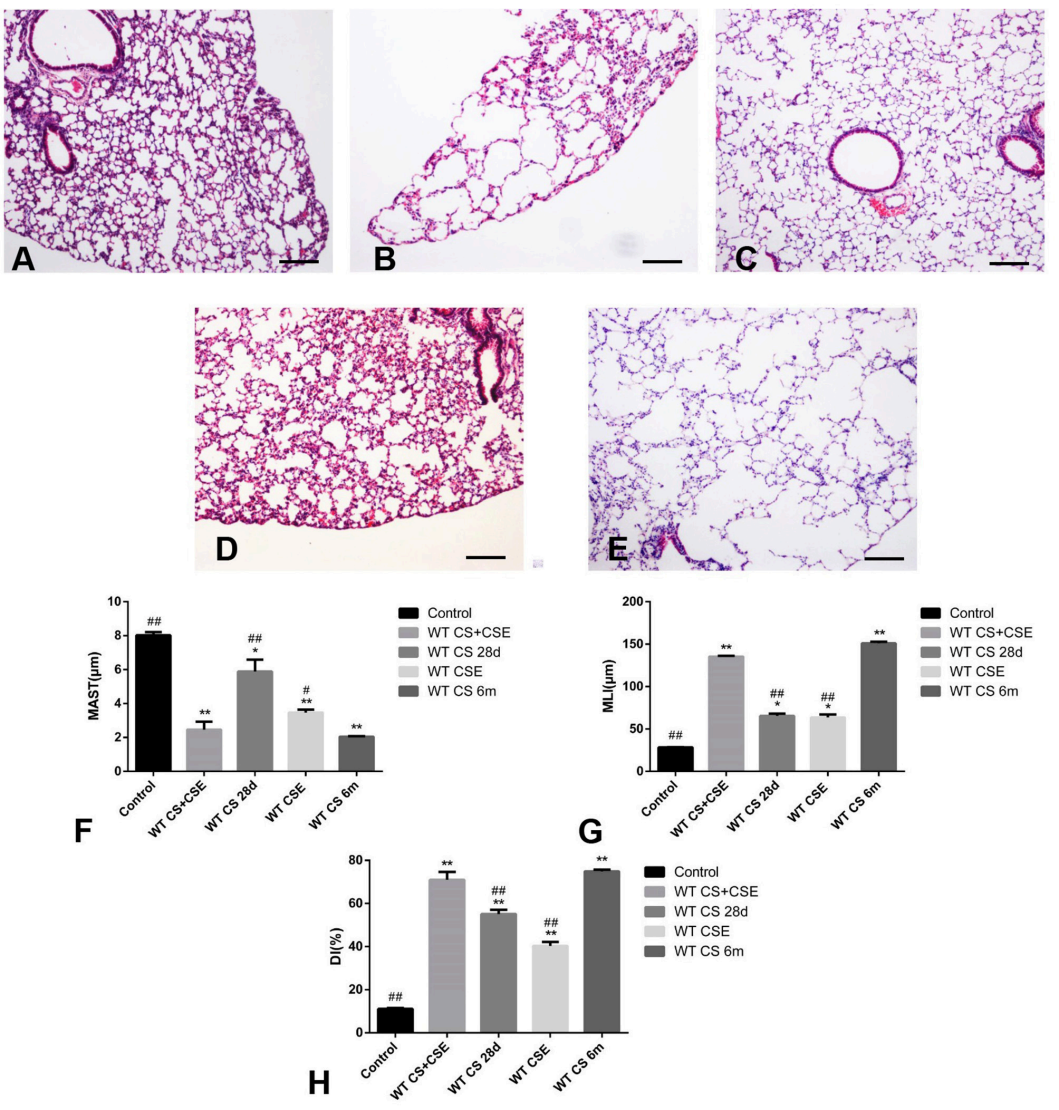
Pathological changes of WT mice from different group. **(A)** (100X), H&E staining of lung tissue from CG. **(B)** (100X), H&E staining of lung tissue from NCM, exhibited enlarged alveolar space, thinner alveolar septum, and destroyed alveolar wall when compared with CG **(A)**. **(C)** (100X), H&E staining of lung tissue from SCSE, also exhibited enlarged alveolar space, thinner alveolar septum, and destroyed alveolar wall when compared with CG **(A)** but lighter in than LCSE **(E)**. **(D)** (100X), H&E staining of lung tissue from CSEII, also exhibited enlarged alveolar space, thinner alveolar septum, and destroyed alveolar wall when compared with CG **(A)** but lighter than LCSE **(E)**. **(E)** (100X), H&E staining of lung tissue from LCSE, also exhibited enlarged alveolar space, thinner alveolar septum, and destroyed alveolar wall when compared with CG **(A)**. **(F)** changes of mean alveolar septal thickness (MAST); **(G)** changes of mean linear intercept (MLI); **(H)** changes of destructive index (DI). ^*^Compared with control group, *p* < 0.05; ^**^compared with control group, *p* < 0.001; ^#^compared with WT CS 6m group, *p* < 0.05; ^##^compared with WT CS 6m group, *p* < 0.001. Scale bars: 200 μm. Error bars: mean ± SEM. *n* = 5.

We evaluated the models on three levels: (1) body weight gain, (2) pathological changes, and (3) lung function. Initially, in the first 28 days, compared with CG, the NCM mice lost some weight at the beginning and gained the minimum weight at the end of modeling period. Mice in SCSE and CSEII both gained less weight than CG, but more than NCM. We also recorded the weight gain in the LCSE mice for 6 months and observed that they had gained weight until the end of the 2nd month and kept a relatively low body weight compared with CG (Figures 1A,B, *p* < 0.05). Further, NCM and LCSE showed the severest pulmonary dysfunction with their RAW increased; Cdyn, PEF, and Ti/Te all decreased compared with the CG. SCSE and CSEII also presented pulmonary dysfunction compared with CG but better than NCM and LCSE. Between NCM and LCSE, these lung function items exhibited no significant differences except for Ti/Te (Figure 1C, *p* < 0.05). Moreover, the pathological changes were in line with changes of lung function, that is, NCM and LCSE exhibited lowest MAST and highest MLI and DI%; SCSE and CSEII better, but still worse than CG (Figures 2A–H, *p* < 0.05).

### Expression level of mir-21 in lungs of WT mice with different modeling methods and its correlation with lung function

**Figure 3:**
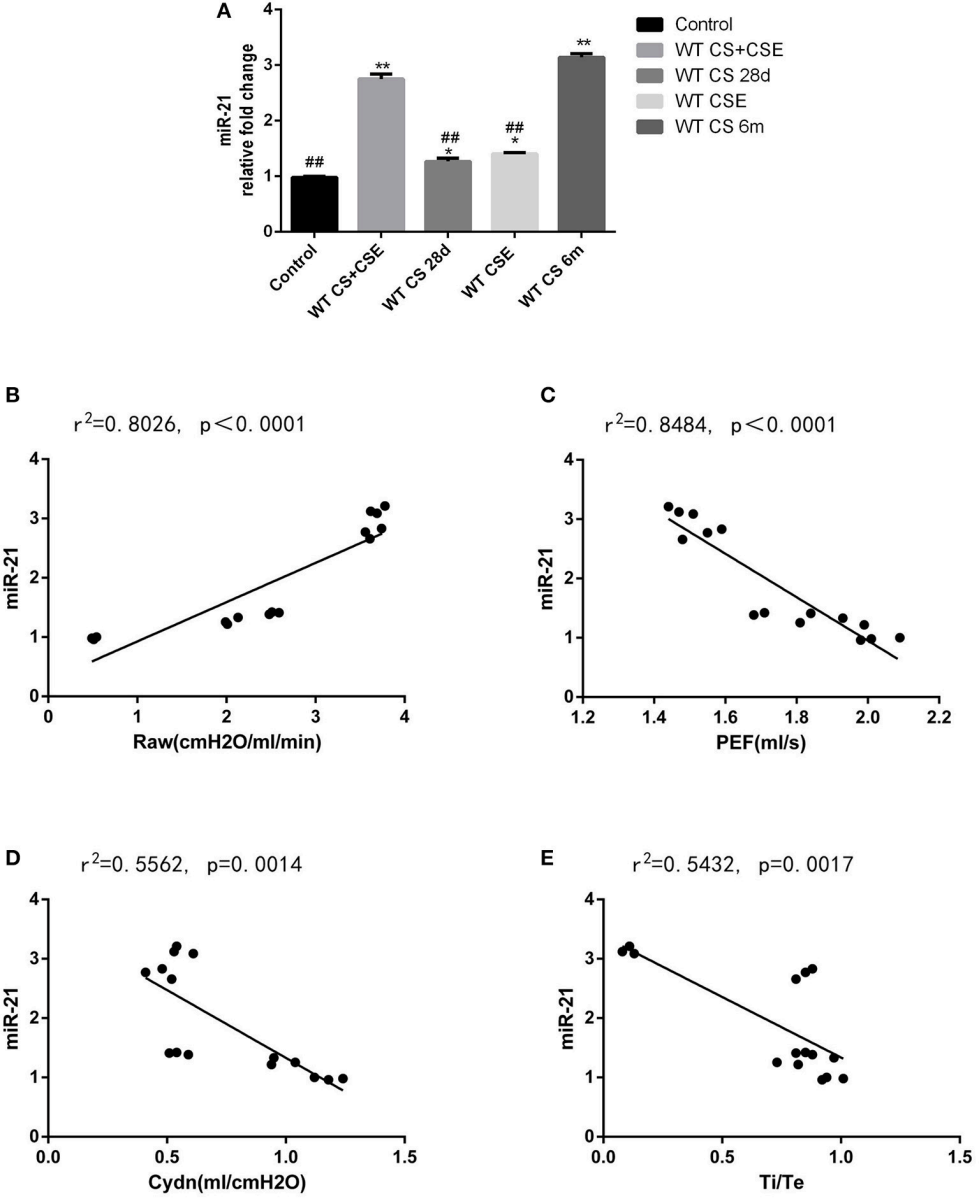
Level of miR-21 in WT mice lungs from different group and the correlation analysis between the level of miR-21 and lung functions. **(A)** Level of miR-21 in WT mice lungs from different group, 2^−ΔΔCT^ method. **(B)** correlation analysis between the level of miR-21, and Airway resistance (Raw); **(C)** correlation analysis between the level of miR-21 and lung dynamic compliance (Cdyn); correlation analysis between the level of miR-21 and peak expiratory flow (PEF); correlation analysis between the level of miR-21 and inspiratory time/expiratory time (Ti /Te). ^*^Compared with control group, *p* < 0.05; ^**^compared with control group, *p* < 0.001; ^#^compared with WT CS 6m group, *p* < 0.05; ^##^compared with WT CS 6m group, *p* < 0.001. Error bars: mean ± SEM. *n* = 5.

Mice in NCM and LCSE had the highest miR-21 level in their lungs, followed by SCSE and CSEII. CG showed the least amount of miR-21 in lung tissue (Figure 3A, *p* < 0.05). According to results of the Pearson analyses, we found the level of miR-21 was positively correlated with RAW but Cdyn, PEF and Ti/Te, negatively (Figures 3B–E, *p* < 0.05).

### The role of mir-21 knock-out in the occurrence of COPD *in vivo*

**Figure 4:**
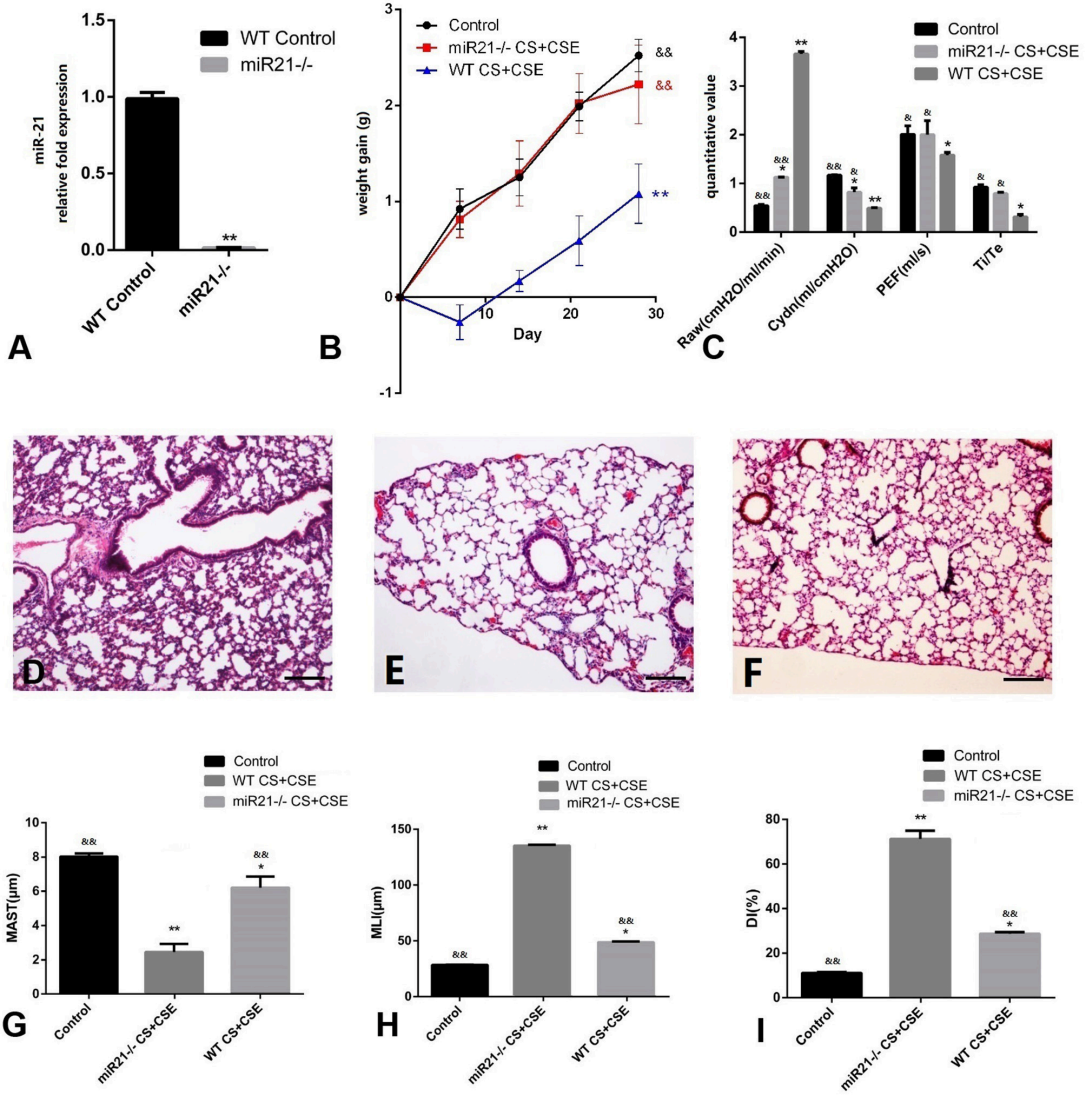
Evaluation of miR-21^−/−^ mice modeled by CS exposure + CSE injection. **(A)** Level of miR-21 in miR-21^−/−^ mice lung. **(B)** Trends of mice body weight gain during modeling days of different group. **(C)** Lung functions of mice in different group. **(D)** H&E staining of lung tissue from GC (100X). **(E)** H&E staining of lung tissue from NCM (100X), exhibited enlarged alveolar space, thinner alveolar septum, and destroyed alveolar wall when compared with CG **(D,F)** H&E staining of miR-21^−/−^ CS+CSE group (100X), exhibited moderately enlarged alveolar space, thinner alveolar septum, and destroyed alveolar wall when compared with CG **(D)**, but lighter than NCM **(E)**. **(G)** Changes of mean alveolar septal thickness (MAST); **(H)** changes of mean linear intercept (MLI); **(I)** changes of destructive index (DI). ^*^Compared with control group, *p* < 0.05; ^**^compared with control group, *p* < 0.001; ^&^compared with WT CS+CSE group, *p* < 0.05; ^&&^compared with WT CS+CSE group, *p* < 0.001. Scale bars: 200 um. Error bars: mean ± SEM. *n* = 5.

Similar to how we evaluated the effects of different modeling methods in the WT mice, we observed the results of miR-21^−/−^ mice modeled by the NCM method and compared the CG and with the NCM. We ran RT-PCR in the lungs of miR-21^−/−^ mice and were hardly able to detect the expression of miR-21 (Figure 4A, *p* < 0.05). Secondly, miR-21^−/−^ mice put on more weight than the NCM mice, but less than the CG mice after modeling (Figure 4B, *p* < 0.05). Further, small changes (both lung function and pathological changes) took place in miR-21^−/−^ mice compared with NCM mice, but still more obvious than CG (Figures 4C–I, *p* < 0.05).

### Inflammatory cells infiltration in lungs of mice modeled by different methods and the effect of mir-21 on this phenomenon

**Figure 5:**
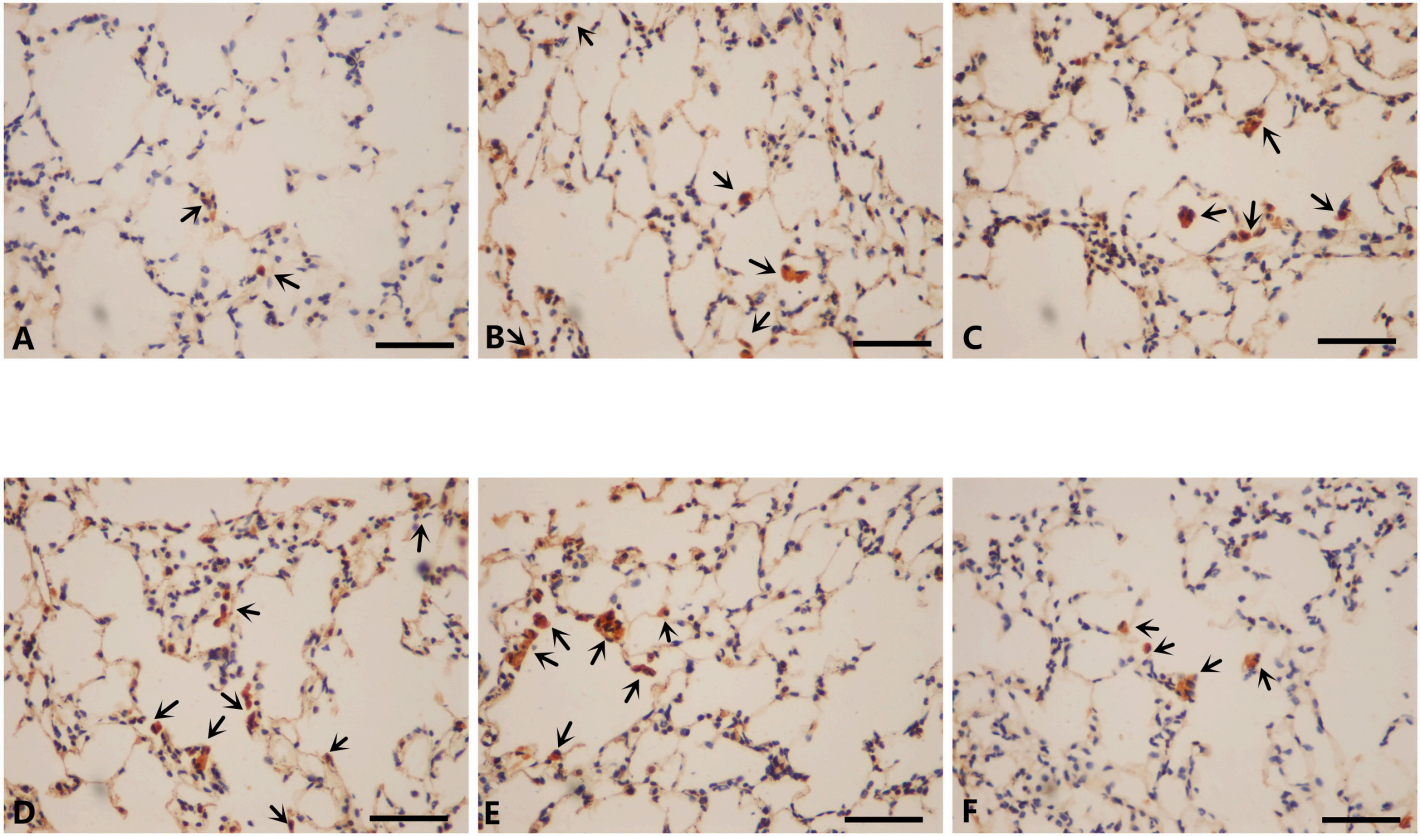
IHS of CD3(400X). **(A)** Contro group. **(B)** WT CS 28d group. **(C)** WT CSE group. **(D)** WT CS+CSE group. **(E)** WT CS 6m group. **(F)** miR-21^−/−^ CS+CSE group. Black arrows: positive cells, scale bar: 50 μm.

**Figure 6:**
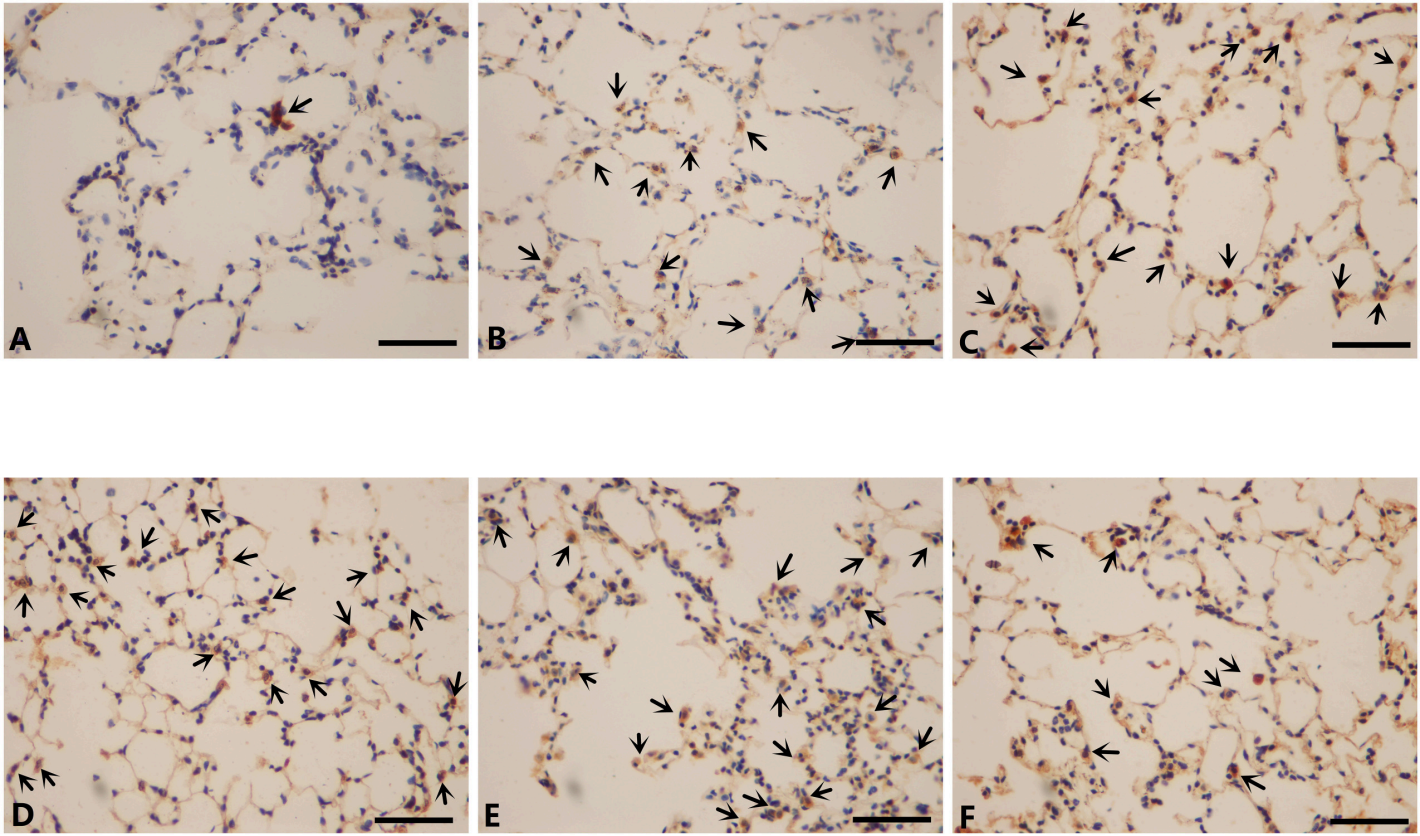
IHS of CD68 (400X). **(A)** Contro group. **(B)** WT CS 28d group. **(C)** WT CSE group. **(D)** WT CS+CSE group. **(E)** WT CS 6m group. **(F)** miR-21^−/−^ CS+CSE group. Black arrows: positive cells, scale bar: 50 μm.

**Figure 7:**
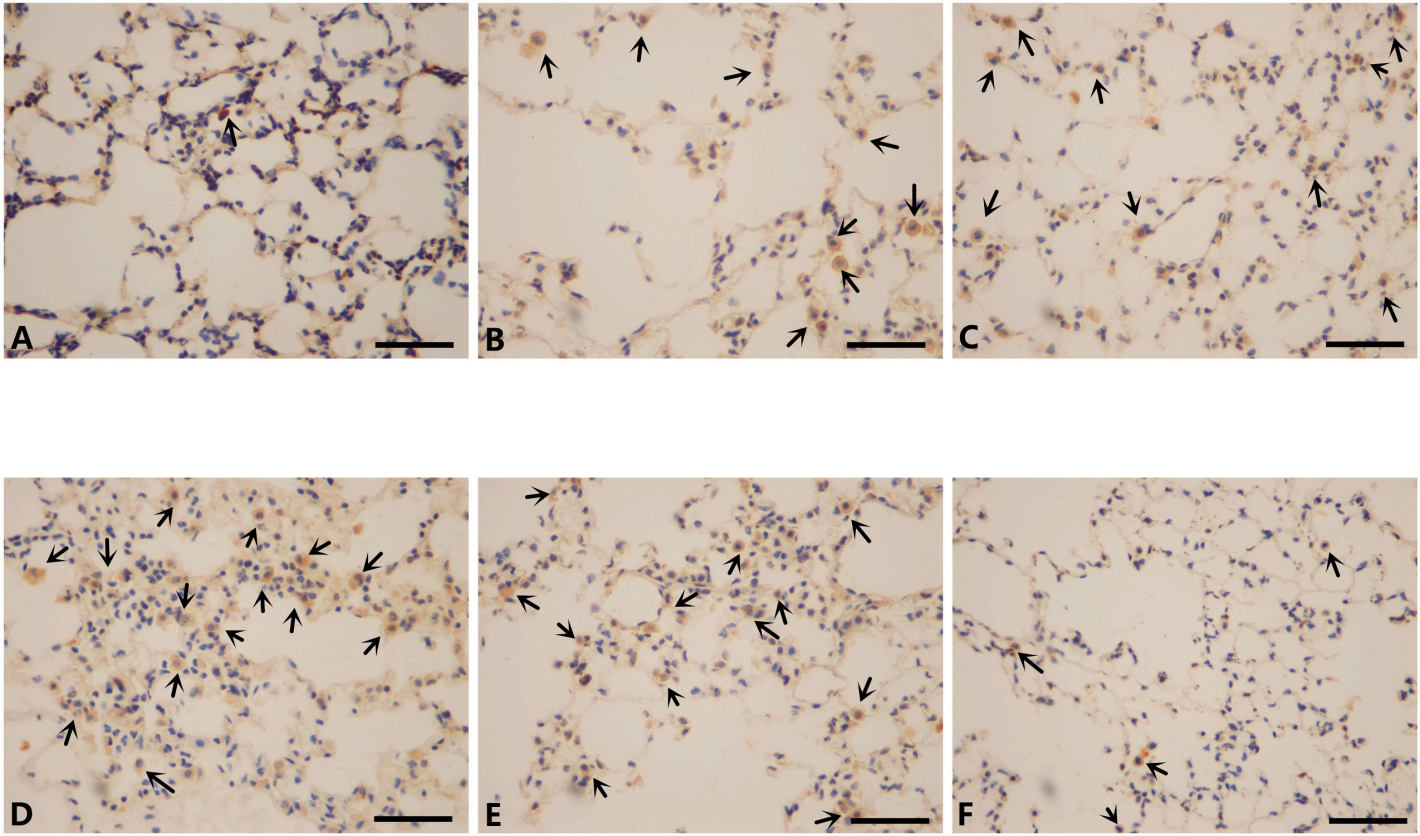
IHS of MPO (400X). **(A)** Contro group. **(B)** WT CS 28d group. **(C)** WT CSE group. **(D)** WT CS+CSE group. **(E)** WT CS 6m group. **(F)** miR-21^−/−^ CS+CSE group. Black arrows: positive cells, scale bar: 50 μm.

**Figure 8:**
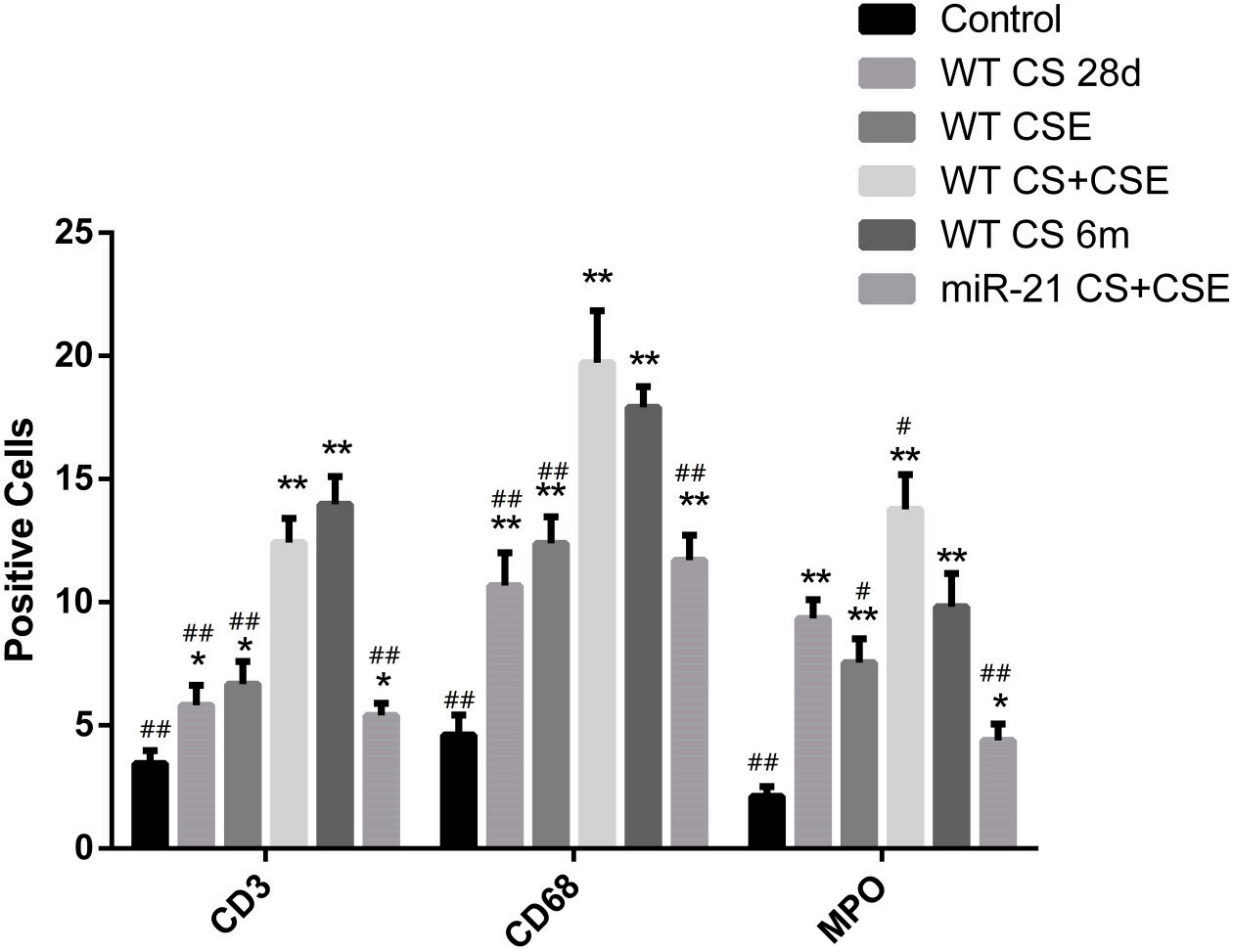
Number of different positive cells in different groups. ^*^Compared with control group, *p* < 0.05; ^**^compared with control group, *p* < 0.001; ^#^compared with WT CS 6m group, *p* < 0.05; ^##^compared with WT CS 6m group, *p* < 0.001. Error bars: mean ± SEM. *n* = 5.

To further investigate the changes induced by different modeling methods and the effect of miR-21 knock-down on it, we thus ran IHS to evaluate the number of several inflammatory cells in lungs of mice from different groups. The number of CD3+ cells (lymphocytes), CD68+ cells (macrophages), and MPO+ cells (neutrophils) were all ascended the maximum in LCSE (Figures 5E, 6E, 7E, 8) and NCM (Figures 5D, 6D, 7D, 8), compared with GC (Figures 5A, 6A, 7A, 8). SCSE (Figures 5B, 6B, 7B, 8) and CSEII (Figures 5C, 6C, 7C, 8), still, had more inflammatory cells infiltration of all of those three cell types than CG but less than LCSE and NCM. Surprisingly, in the lungs of modeled miR-21^−/−^ mice (Figures 5F, 6F, 7F, 8), the number of those inflammatory cells were all less than LCSE and NCM, but a little bit more than CG.

## Discussion

In the present study, we initially proposed a novel COPD modeling method—Intraperitoneal injection of CSE combined with CS exposure for 28 days—which showed similar results compared with LCSE (Wright et al., 2008; Givi et al., 2013). In addition, we applied another method (CSEII) reported previously (Zhang et al., 2013), along with a short-term CS exposure method. We detected the expression level of miR-21 in lungs of all WT mice and found some increases at varying levels after modeling. Accordingly, we introduced the miR-21^−/−^ mice into the next experiment to define its role in COPD. To further confirm the results, we next ran IHS to observe the situation of inflammatory cells infiltration in lungs of all the mice enrolled in this study.

The long-term CS exposure method is widely accepted for mice COPD models and has remarkable results. However, this method is time consuming and we, therefore, designed a novel method to improve modeling efficiency. As was reported previously, some researchers established the emphysematous by CSE intraperitoneal injection (Chen et al., 2009) and further experiments have been conducted to confirm the effectiveness of this method (He et al., 2015). The mechanism in this method is not completely known, But is thought to be involved with autoimmune mechanisms in alveolar septal cell destruction (Agustí et al., 2003; Taraseviciene-Stewart et al., 2006); this method was not sufficiently clinically relevant. We therefore designed a novel method which is more related to the clinical cause of COPD by adding a CS exposure process with some modifications. The results confirmed this method is almost equivalent to the long-term CS exposure method and better than the CSE intraperitoneal injection only method in four aspects—lower body weight gain, emphysematous changes in lung, lung function deterioration and an increased level of inflammation.

In general, the changes in COPD are both pathological and pathophysiological, that is, emphysema and small airway disease in lungs as well as a progressive lung function deterioration (Wright et al., 2008), and additionally, the weight loss (muscle mass loss of the quadriceps muscle; Jones et al., 2015). The golden assessment standard of an animal model regarding a certain disease is whether the model can reproduce the changes according to the corresponding disease as much as possible. We therefore measured the results of different methods in those aspects, which are lung function, pathological changes in lungs and the infiltration of inflammatory disease as well as trends of body weight during modeling.

As were found by us, in the first 28 modeling days, our novel method led to the least weight gain compared with the other groups. And it was from the end of the 2nd month when LCSE stopped putting on weight and ended up with a relatively low body weight compared with CG and this result is in line with our previous study (Lu J. J. et al., 2017). Regarding to the lung function of mice, LCSE showed the severest deterioration among all the groups, and that is why this method is the most acceptable and advocated one worldwide. However, NCM also showed a fairly remarkable lung function decrease, worse than the rest of those groups. SCSE and CSEII also both exhibited the same trend in lung function as our method but not that obvious. Pathologically speaking, LCSE exhibited the most emphysematous and inflammatory changes. NCM could lead to many pneumatoceles in a short time, along with some inflammatory changes to a certain extent. SCSE could also cause some pneumatoceles but less inflammation changes, and CSEII the other side, with some inflammatory change but less emphysema changes. It might result from that CSE injection could cause a “systematic inflammatory response” in body immediately as mentioned above (Fabbri and Rabe, 2007) and CS exposure could directly do harm to the airways and alveolus, resulting in obvious structural changes like emphysema. These results indicated our newly designed method led to fairly ideal COPD-related changes, just like what occurred in the real COPD patients, and notably, more efficient and economical.

As we previously reported that miR-21 expressed differently in the serum of healthy people, smokers and COPD patients, suggesting it could be related to the occurrence of COPD and a potential indicator of COPD severity (Xie et al., 2014). To further investigate its effectiveness as an indicator, we thus ran RT-PCR to detect the level of miR-21 in lungs of mice from different groups as they had COPD to different extents. Just like what we assumed before, the level of miR-21 in lungs was detected the highest both in LCSE and NCM, followed by the SCSE and CSEII. And they were all higher than CG. This trend was further confirmed by the correlation analyses, suggesting miR-21 is negatively correlated with lung function. Moreover, when we modeled the miR-21^−/−^ mice by our novel method, surprisingly, we found the knock-down of miR-21^−/−^ played a protective role in COPD. In another word, miR-21 might be a promoting gene in COPD. This find was in line with Ong et al. (2017), who argued miR-21 could give rise to TGF-β and finally leading to lung tissue repairment and remodeling which might eventually cause persistent and irreversible small airway obstruction. Nevertheless, we have to admit that knocking down of miR-21 did not reverse the COPD-related changes completely. It might because COPD is a multi-pathogenesis disease and the unusually increased miR-21 level is just one of many pathogenic factors. However, it might work independently to some extent in COPD.

The persistent chronic inflammation is another feature of COPD (Gautam and O'Toole, 2016). To reconfirm that, we ran IHS on lung paraffin sections and found there were a massive filtration of lymphocytes, macrophages and neutrophils, in line with many previous studies (Baraldo et al., 2004; Brightling and Pavord, 2004; Olloquequi et al., 2010). And moreover, Zhao et al. (2018) even advocated this could be a special phenotype of COPD, deserving extra attention in treatment. Further, many studies also reported miR-21 could be a promoter of inflammation in not only pulmonary diseases but also other systems (Shi et al., 2013; Chen et al., 2017; Lee et al., 2017; Venugopal et al., 2017), when we tested the lung sections from miR-21^−/−^ mice, we found by IHS the number of all those inflammatory cell infiltration are less than either LCSE and NCM. According to Kupczyk and Kuna (2014), miRs could not only be biomarkers for respiratory diseases but also potential treatment target. Along with our previous and present studies, it is reasonable to speculate blocking the function of miR-21 could be a potential treatment for COPD. However, more research is necessary to prove such potential treatment.

Some limitations in the present study merit consideration. First, we enrolled 50 WT mice in total with 10 mice allocated to each group. When the modeling methods were complete, only five mice from each group underwent lung function tests due to certain technical reasons, including imperfect anatomy skills and failed lung function test processes. To ensure consistency across all of the data, we collected for analysis the lung tissue of mice who underwent a complete lung function test. This issue could potentially lead to some bias in the final conclusions reached. Second, the method of CSE intraperitoneal injection is not clinically relevant, making the model not a complete duplicate of a real COPD patient. However, CSE intraperitoneal injection is a very safe and convenient method to carry out, and its purpose is to make the toxic materials absorbed into blood just like when humans absorb those toxic materials through capillaries in lungs when they are smoking. Therefore, we think this method is relatively effective and efficient and the results of the present study had also proved it.

## Conclusion

To summarize, this CSE intraperitoneal injection combined with short-term CS exposure COPD modeling method in mice is more efficient and economical than other methods including the long-term CS exposure one, and miR-21 could be a practical indicator for COPD severity. Moreover, blocking the function of miR-21 could be potential treatment of COPD.

## Author contributions

The article was written by SH and revised by LX. The study was designed by LL, LX, and performed by SH, LL, and ZZ. Statistical analysis was performed by SH and LL. Technical supports were offered by JL, ZZ, SS, and LX. Research fund was offered by LX and SS.

### Conflict of interest statement

The authors declare that the research was conducted in the absence of any commercial or financial relationships that could be construed as a potential conflict of interest.
